# Molecular characteristics of *Staphylococcus aureus* strains isolated from nasal samples of sixth year medical students during their pediatric services practices

**DOI:** 10.1186/s12941-021-00429-8

**Published:** 2021-04-17

**Authors:** Kamile Arıkan, Eda Karadag-Oncel, Emre Aycan, Banu Sancak, Mehmet Ceyhan

**Affiliations:** 1 Department of Pediatric Infectious Diseases, Izmir Behcet Uz Children’s Hospital, Health Sciences University, Izmir, Turkey; 2 Department of Pediatric Infectious Diseases, Izmir Tepecik Research and Training Hospital, Health Sciences University, Izmir, Turkey; 3 Department of Pediatric Infectious Disease, Hacettepe University Faculty of Medicine, Ankara, Turkey; 4 Department of Medical Microbiology, Hacettepe University Faculty of Medicine, Ankara, Turkey

**Keywords:** Methicillin resistant, *Staphylococcus aureus*, Nasal carriage, Panton Valentine Leukocidin, Staphylococcal cassette chromosome *mec* (SCC*mec*) types, Medical students, Pediatric services

## Abstract

**Background:**

Methicillin-resistant *Staphylococcus aureus* (MRSA) strains are prevalent in healthcare services. Medical students are at risk for MRSA carriage, subsequent infection and potential transmission of nosocomial infection.Few studies have examined MRSA carriage among medical students.

**Methods:**

In this prospective cohort study, between July 2016 and June 2017, two nasal swab samples were taken per student 4 weeks apart during their pediatric internship. MRSA typing was performed by staphylococcal cassette chromosome *mec* (SCC*mec*) types, Panton Valentine leukocidin (PVL) encoding genes.

**Results:**

A total of 239 sixth year medical students, 164 (68.6%) male (M/F:2.1),with median age 25 years (min–max; 23–65 years) were included in this prospective cohort study. Among 239 students, 17 students (7.1%) were found to be colonized with methicillin-sensitive *S. aureus* (MSSA) at the beginning of pediatric internship. After 4 weeks, at the end of pediatric internship totally 52 students were found to be *S. aureus* colonized (21.8%). Three of 52 *S. aureus* isolates were MRSA (1.3%) and the rest was MSSA (20.5%), all were PVL gen negative. Two of three MRSA isolates were characterized as SCC*mec* type IV, one isolate was untypeable SCC*mec*. Nasal carriage of *S. aureus* increased from 7.1% to 21.5% (p < 0.001). Nasal *S. aures* colonization ratio was higher in students working in pediatric infectious disease service (p = 0.046). Smoking was found to be associated with a 2.37-fold [95% CI (1.12–5.00); p = 0.023] and number of patients in pediatric services was 2.66-fold [95% CI (1.13–6.27); p = 0.024] increase the risk of nasal *S. aureus* colonization. Gender was not found to increase risk of MRSA carriage.

**Conclusion:**

MSSA nasal carriage increased at the end of pediatric internship and significantly high in students working in pediatric infectious diseases services. Smoking and high number of patients in pediatric services significantly increase *S.aureus* colonization.

## Introduction

*Staphylococcus aureus (S.aureus)* remains a common cause of infection in the community for centuries. The spectrum of the diseases caused *S. aureus* varies from mild skin infection to endocarditis or life-threatening septicemia. In the last few decades, methicillin-resistant *Staphylococcus aureus* (MRSA) has become an increasingly important cause of healthcare-associated infections.

Methicillin-resistant *S. aureus* widely known as the superbug, was first reported in the early 1960s. Within years, it rapidly spread in the community and health-care settings. At present, MRSA is endemic in health-care settings globally, which results in a heavy disease burden. MRSA infections were associated mostly with hospitals and infected patients who had contact with the health-care system, hence the name health-care associated MRSA (HA-MRSA) [1–4]. These strains were typically resistant to multiple antimicrobials and carried large staphylococcal cassette chromosome *mec* (SCC*mec*) elements, mostly types I–III. Cases of MRSA infections have been increasingly reported during the last decade in healthy individuals of the general population with no risk factors for these community-associated MRSA (CA-MRSA) infection. Community-associated MRSA strains were not multidrug-resistant, carried small SCC*mec* elements, mostly types IV and V, and also carried the genes encoding the Panton Valentine leukocidin (PVL), a pore-forming toxin [5–7].

Health care workers (HCWs) play a significant role in the epidemiology of MRSA infections.HCWs act as vectors for transmission of MRSA as they work at the interface between hospital and the community. Transmission of MRSA in the healthcare environment is usually by contact between patients through hands, clothes or equipment of HCWs.

Dynamics and prevalence of MRSA colonization are affected by several factors such as type of hospital department, MRSA prevalence among patients, and inadequate compliance with infection control measures; these differ depending on geographical locations [8–10].

Medical students represent an important portion of the health-care personnel, and they are in frequent contact with susceptible patients; thus, they are at risk of being colonized with *S. aureus*, and of spreading them to susceptible patients. The aim of this study was to examine *Staphylococcus aureus* nasal carriage, SCC*mec* and PVL molecular typing and risk factors of medical students during their pediatric services practices.

## Material and methods

The study was performed at Hacettepe University Faculty of Medicine, between July 2016 and June 2017 in sixth year medical students working in pediatric services; neonatal intensive care unit, neonatal surgery service, intensive care unit, infant service, young child service, adolescent service, hematology service, infectious disease service during 4 weeks internship.

Students who agreed to participate were asked to sign an informed consent and complete a written questionnaire on demographics and medical history. Variables included in the questionnaire were age, gender, housemate, living with children and/or health-care personnel, history of infections, allergies, chronic underlying diseases, smoking habits, antibiotic usage in the previous three months, surgeries and hospitalizations in the previous 1 year. Exclusion criterias were usage of intranasal mupirocin, oral beta-lactam antibiotic and/or clindamycin in the past 3 month. Also students working with other health care workers were excluded.

The study was approved by the Ethics Committee of Hacettepe University, Faculty of Medicine.

### Isolation, growth and identification of bacteria

Samples were collected from both anterior nares (vestibulum nasi) using nasal swabs with a standard rotating technique from sixth year medical students, four weeks apart; at the beginning and at the end of abovementioned pediatric services. Swabs were taken during the admission process, were transported in Stuart’s medium, stored at 4 °C and plated within a few hours on Columbia and Mannitol Salt Agar plates (all Oxoid, Basingstoke, UK) and incubated at 37 °C for 24 h. Colony characteristicson the culture plates and Gram-staining were usedto further confirm the identity of *S. aureus.* Salt tolerance and mannitol fermentation properties of *S. aureus* result in the typical yellow colonies due to a changein the pH. Gram staining helped to ascertain thatthere were no other airborne contaminants by con-firming the characteristic morphology of *S. aureus.* Yellow colonies were selected and confirmed to be *S. aureus* following catalase, coagulase, and DNAse tests and were finally confirmed by PCR using specific primers as shown below. ATCC 25923 standart strain was used for culture and biochemical reactions, *S. aureus* ATCC 29213 standart strain was used for antimicrobial susceptibility tests. *S. aureus* ATCC 49775 standart strain was used for PVL gen PCR analysis. Standart strains for SCC*mec* PCR analysis were as follows respectively; *S.aureus* COL for SCC*mec*I, *S.aureus* BK2464 for SCC*mec* II, *S.aureus* ANS46 for SCC*mec* III, *S.aureus* HDE288 for SCC*mec* IV.

Tests for methicillin resistance were performed using the Kirby-Bauer disc diffusion method, using cefoxitin (30 μg) disc on Mueller–Hinton agar with 24-h. Results were interpreted according to the criteria of CLSI (2017). Zone of inhibition of cefoxitin disc ≥ 22 mm was accepted as methicillin-sensitive *S. aureus.*

The *mecA* gene, SCC*mec* types and PVL genes were detected by polymerase chain reaction (PCR). MRSA isolates were subjected to SCC*mec* typing as described by Oliveira et al. [11], which is based on a set of multiplex PCR reactions with 14 primers. SCC*mec* types I–IV were assigned according to the combination of the cassette chromosome recombinase (ccr) type and *mec* class. MRSA isolates that could not be assigned to any expected type were defined as non-typable (NT).

## Statistical analysis

All statistical analyses were performed using the SPSS package program for Windows, version 17.0 (SPSS Inc., Chicago, IL, USA). Values for numerical variables were provided as mean ± standard deviation or median (minimum–maximum) depending on normality of distribution. Categorical variables were provided as absolute values or percentages, the comparisons of which were made using the chi-square test. Two-way comparisons for numerical variables were made using the Mann–Whitney U test, whereas the Kruskal–Wallis test was used for comparison involving more than two groups. Factors associated with an increased nasal *S. aureus* colonization were identified using logistic regression analysis. A p-value of < 0.05 was considered indicative of statistical significance.

## Results

A total of 239 sixth year medical students, of whom 164 (68.6%) male, were included in this prospective cohort study. Median age of the students was 25 years (min–max; 23–65 years). Eight (3.3%) of the students were married, 116 (48.5%) students were living friends. Twenty-two (9.2%) students were living with at least one child. Underlying diseases of students that may increase risk of S*. aureus* colonization were; allergic rhinitis (n = 30) and diabetes mellitus (n = 1). Seventy-one students (29.7%) were occasionally smoker, 20 students (8.4%) were frequent smoker. None of the students had history of hospitalization or surgery history at the previous one year (Table 1).

**Table 1:** Sociodemographic characteristics of sixth year medical students working in pediatric services

Characteristics	n = 239
Age (years)^a^	25 (23–65)
Gender^b^	
Male	164 (68.6)
Marital status^b^	
Married	8 (3.3)
Medine education duration^b^	
6 years	161 (67.4)
> 6 years	78 (32.6)
Living with a child at home^b^	22 (9.2)
Smoking status^b^	
None smoker	148 (61.9)
Occasional smoker	71 (29.7)
Frequent smoker	20 (8.4)
Chronic disease^b^	
Allergic rhinitis	30 (12.6)
Diabetes mellitus	1 (0.4)
Antibiotic usage in the past 3 months^b^	27 (11.3)
Infection history in the past 3 months^b^	
Upper respiratory tract infection	68 (28.5)
Skin infection	5 (2.1)

^a^Values are given median and min–max

^b^Values are given as percentage

At the beginning of pediatric internship, 44 students (18.4%) were working in child intensive care unit, 42 students (17.6%) were working in infant infectious diseases service, 37 students (15.5%) were working in pediatric infectious diseases service, 33 students (13.8%) were working in neonatal surgery service, 32 students (13.4%) were working in hematology service, 22 students (9.2%) were working in young child service, 22 students (12.1%) were working in adolescent service. *S. aureus* was cultured from 17 of 239 (7.1%) nasal cultures taken at the beginning of pediatric internship, all of the 17 (7.1%) isolates were methicillin sensitive, PVL gen negative. Sixteen of 17 MSSA cultured students (94.1%) were not married, two students (11.8%) were living alone, six students (35.3%) were living with family, nine students (52.9%) were living with friends, eleven students (64.7%) were on medical education for more than 6 years, six students (35.3%) were frequent-smokers, two students (11.8%) stated using steroid in the past 3 months, three students (17.6%) stated using antibiotics in the past 3 months, eight students (47%) explained of having respiratory tract infections in the past 3 months. None had history of hospitalization in the past 3 months.

At the end of pediatric intership, *S. aureus* was isolated in 52 of 239 (21.8%) nasal sample cultures; 49 (20.5%) MSSA, 3 (1.3%) MRSA (Table 2). Two of three MRSA (66.7%) cultured nasal samples were taken from students working in infant infectious diseases service, one was from student (33.3%) working in pediatric infectious diseases service.None of the 52 *S. aureus* isolates were PVL gen positive. Two of three MRSA isolates were SCC*mec* tip IV (66.6%), one MRSA isolate was non-typeable. One of 49 MSSA cultured students was married (2%), 31 students (63.3%) were living with friends, eleven students (22.4%) were living with family, seven students (14.3%) were living alone, two students (4.1%) were living with a child at home. Twenty students (40.8%) were on medical education for more than six years, 26 students (53.1%) were frequent-smokers, three students (6.1%) stated using steroid in the past three months, eight students (16.3%) declared using antibiotic in the past three months, eighteen students (36.7%) had history of respiratory tract infection in the past three months. Two of three MRSA isolated students (66.7%) stated living with health-care worker at home, were on medical education for more than six years. One of three MRSA isolated student (33.3%) stated frequent smoking, upper respiratory tract infection history in the past three months. None of three MRSA isolated students were married nor had history of using antibiotics or steroid in the past three months.

**Table 2:** Nasal *Staphylococcus aureus* carriage results taken at the first day and the last day of pediatric internship

Sampling time	Culture results (n = 239)			
	Negative	MSSA	MRSA	Total positivity
First sample	222 (92.9)	17 (7.1)	–	17 (7.1)
Second sample	187 (78.2)	49 (20.5)	3 (1.3)	52 (21.8)

*MSSA* methicillin sensitive *Staphylococcus aureus*

*MRSA* methicillin resistant S*taphylococcus aureus*

**Fig. 1:**
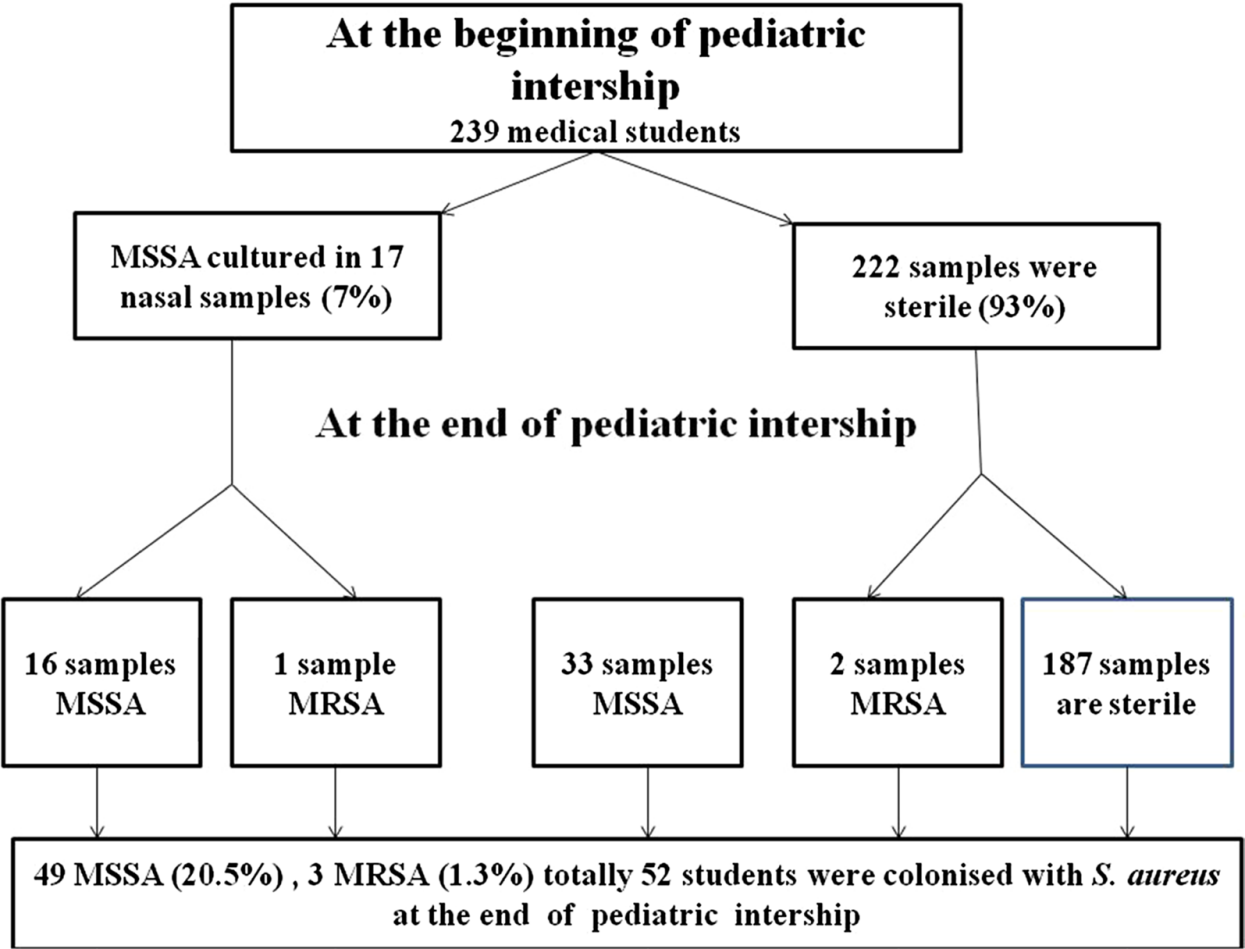
Flow chart of the study

**Fig. 2:**
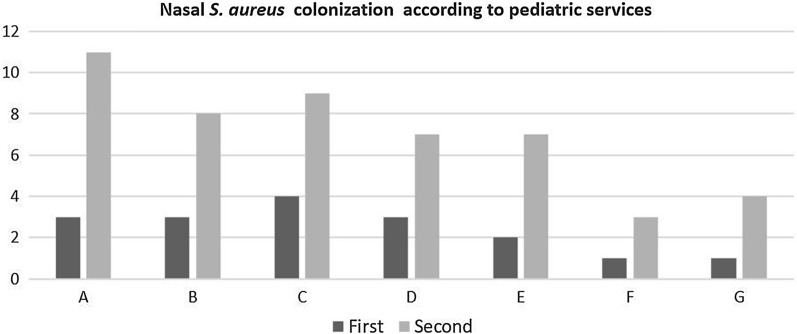
Nasal *S. aureus* colonization according to pediatric services at the beginning and at the end of the internship

Increase in nasal colonization of *S. aureus* in comparison with first and second samples was statistically significant (p < 0.001). Sixteen of 17 MSSA isolates cultured at the first samples were found MSSA again at the second samples, but 1 MSSA isolate cultured at the first samples was found as MRSA at the second sample (Fig. 1). Nasal *S. aureus* colonization according to pediatric services at the beginning and at the end of the internship was depicted in Fig. 2. Nasal S*. aureus* colonization at the end of infectious disease service internship was statistically significantly increased (p = 0.046).

It was found that living alone or with family, friends did not increase the risk of nasal *S. aureus* colonization in students (p = 0.55). Living with a child (p = 0.177) and/or history of using antibiotic (p = 0.457) and/or steroid (p = 0.731) in the past three months, history of upper respiratory tract or skin infections in the past three months did not increase the risk of nasal *S. aureus* colonization (p = 0.138).

Smoking was found to be associated with a 2.37-fold [95% CI (1.12–5.00); p = 0.023] and number of patients in pediatric services was 2.66-fold [95% CI (1.13–6.27); p = 0.024] increase the risk of nasal *S. aureus* colonization (Table 3).

**Table 3:** Multivariate analysis for increasing nasal S. aureus colonization

Variables	Odds ratio (95% CI)	*p*
Number of patients in pediatric services	2.655 (1.136–6.270)	0.024
Smoking	2.374 (1.127–5.000)	0.023

*CI *confidence interval

## Discussion

To the best of our knowledge, this is the first study to report nasal *S. aureus* and MRSA carriage rates, molecular characteristics in medical students, according to services at the beginning and end of their pediatric practices. In addition, this is the first study from Turkey in which PVL and SCC*mec* gens were studied from *S. aureus* nasal samples of medical students. Among 239 students, 17 students (7.1%) were found to be colonized with MSSA at the beginning of pediatric internship. After four weeks, at the end of pediatric internship totally 52 (21.5%) students were found to be *S. aureus* colonized. Three of 52 (1.3%) *S. aureus* isolates were MRSA and the rest was MSSA (20.5%). Two of three MRSA isolates were characterized as SCC*mec* type IV, one isolate was untypeable SCC*mec*. Nasal carriage of S*. aureus* increased statistically significantly from 7.1% to 21.5%. Nasal *S. aures* colonization ratio was higher in students working in pediatric infectious diseases service. Smoking and number of patients in pediatric services was found to increase risk of nasal S*. aureus* colonization.

In a study conducted in Poland, asymptomatic colonization with *S. aureus* strains was found in 245/955 (25.7%) students, in particular years in the range of 21.7–29.9%, similar to our results but molecular analysis was not done in aforementioned study [12]. In a study from Israel, among 58 students, *S. aureus* carriage rates increased from 33 to 38% to 41% at baseline (preclinical studies), 13 and 19 months (clinical studies), respectively (p = 0.07). MSSA carriage increased in the clinical studies period (22 to 41%, p = 0.01), similar to our results. Overall, seven students (12%) carried 13 MRSA isolates. MRSA isolates were PVL negative and were characterized as SCC*mec*II-t002, SCC*mec*IV-t032, or t12435 with untypable SCC*mec*. In our results, MRSA carriage was not as high as this study also SCC*mec*IV was the dominant type [13]. In another study from China conducted in interns, the prevalence of *S. aureus* among staphylococcal specimens was 23.1%, similar to our results, and in that study the MRSA prevalence was reported as 9.4% with SCC*mec* type IV dominancy, PVL gen positivity in 10 (45.4%) MRSA isolates [14].

In a study conducted from Turkey in 269 health care workers, the prevalence of *S. aureus* carriage was 20.1%, only one MRSA (0.37%), similar to our results. In that study, *S. aureus* carriage was found to be significantly lower in the smoker group (p = 0.015) in opposite to our results [15]. In another study from Turkey, nasal samples from elementary school children were taken and all MRSA isolates by harbored the SCC*mec* type IV element, but not the PVL gene like in our results [16]. In our study, nasal *S. aureus* colonization of medical students was 7.1% at the beginning of pediatric internship. In second samples, 3 MRSA (1.3%) and 49 MSSA (20.5%) totally 52 students (21.8%) were colonized with *S. aureus* isolates. Two of 3 MRSA isolates were harboring SCC*mec* type IV gene. None of the *S. aureus* isolates were harboring PVL gene. SCC*mec* type IV and PVL gens are known to be more common in community-acquired MRSA infections. Our results support possibility of SCC*mec* type IV positivity in hospital-acquired MRSA isolates. In a study conducted in hospitalized adult patients between 2004 and 2005 in Hacettepe University, 110 MRSA isolates were found to be *mecA* gen positive, 68 MRSA isolates were harboring (61.8%) SCC*mec* type III, 38 isolates (34.5%) were harboring SCC*mec* IIIB and 3 isolates (2.7%) were harboring SCC*mec* tip IV [17]. In another study conducted in our center in hospitalized pediatric patients, 2 of 30 MRSA (6.7%) isolates was were found to be positive for SCC*mec* type III and 16 MRSA isolates (53.3%) were found to be positive for SCC*mec* type IV, indicating SCC*mec* type IV dominancy in our pediatric center [18].

In students working in infant infectious diseases and pediatric infectious diseases service nasal *S. aureus* carriage and risk of methicillin resistance was statistically significantly increased. Nasal S*. aureus* colonization of students was more prevalent in services where the number of hospitalized patients increased. Marital status, living with children or health-care worker or family were not found to increase risk of nasal *S. aureus* colonization. In a study published at Lancet in 2014, nasal *S. aureus* carriage 34.6% in 347 preclinical and clinical medical students, MRSA colonization was 1.9%. Nasal *S.aureus* carriage rate was more than what we found, but in aforementioned study there was no discrimination according to pediatric services as we did [19]. In another study published in 2018, 200 medical students were included, MRSA colonization was 2%, SCC*mec* type I and III were more prevalent [20]. Collazos et al. found that, nasal *S. aureus* colonization rate was 23.6% in 216 medical students and MRSA colonization was 14.3%, compared to our study it was more prevalent [21].

In Turkey MRSA had been noticed as an important nosocomial infection reason since 1980s. Medical students constitute a commonly forgotten health-care workers who are often not as well informed as other health-care personnel about standard infection control measures. As a result of our study, students that had worked in infectious diseases services where patients were hospitalized due to pneumonia, osteomyelitis, septic arthritis, bacteremia, cellulitis, meningitis were more commonly colonized with *S. aureus*, particularly MRSA at the end of pediatric internship. This finding highlighted us about increasing infectious control precautions in these services. It may be logical to check health-care workers for nasal *S. aureus* colonization periodically in services where high risk patients for invasive *S. aureus* infections are hospitalized.
